# Clinical and Environmental Plasmids: Antibiotic Resistance, Virulence, Mobility, and ESKAPEE Pathogens

**DOI:** 10.3390/antibiotics15010029

**Published:** 2025-12-31

**Authors:** Célia P. F. Domingues, João S. Rebelo, Francisco Dionisio, Teresa Nogueira

**Affiliations:** 1 INIAV—Instituto Nacional de Investigação Agrária e Veterinária, 4485-655 Vairão, Portugal; celiapfd@hotmail.com; 2 cE3c—Centre for Ecology, Evolution and Environmental Changes & CHANGE, Global Change and Sustainability Institute, Faculdade de Ciências, Universidade de Lisboa, 1749-016 Lisboa, Portugal; joaorebelo_4@hotmail.com

**Keywords:** plasmids, database bias, plasmid mobility and horizontal gene transfer, metagenomics, antimicrobial resistance genes, virulence genes, ESKAPEE pathogens

## Abstract

Background/Objectives: Plasmids are autonomous DNA molecules that can replicate independently and transfer horizontally between bacterial cells. They play a key role in disseminating adaptive traits, such as antimicrobial resistance and virulence. Our study investigates the fundamental differences between plasmid populations originating from clinical/isolates and environmental/metagenomes. Methods: We compare three distinct plasmid genome datasets—the NCBI Reference Sequence Database (RefSeq), the Integrated Microbial Genomes & Microbiomes system (IMG/PR) from bacterial isolates (I) and microbiomes (M)—to assess how plasmid origin shapes their characteristics, including mobility types, antimicrobial resistance genes (ARGs), virulence genes (VGs) and host taxonomy. Results: We show that plasmids originating from bacterial isolates, more enriched in clinical samples, are fundamentally distinct from recovered from metagenomic data. Plasmids from isolates are larger, enriched in conjugative plasmids and display a higher frequency of ARGs and VGs than the ones assembled from metagenomes. Furthermore, ARGs are more frequently associated with highly mobile plasmids, particularly pCONJ. Conclusions: These findings highlight the importance of plasmid origins in studies of plasmid epidemiology, functional potential and mobility.

## 1. Introduction

Plasmids, extrachromosomal self-replicating genetic elements, are ubiquitous in bacteria. For example, a recent study identified a specific plasmid, *pBI143*, as the most prevalent and abundant genetic element in human gut microbiomes of industrialized populations worldwide [1]. Moreover, studies involving a few thousand strains have shown that there are, on average, two to three different plasmids per strain of *E. coli*, *Klebsiella* and *Salmonella*, as well as almost one plasmid per strain of *Staphylococcus aureus* [2,3]. Plasmids have been extensively studied because they are major drivers of horizontal gene transfer with evolutionary and ecological impact [4], very often may harbor genes that facilitate the degradation of environmental pollutants [5] and biofilm formation, as well as confer bacterial virulence, drug, and heavy metal resistance [6,7,8].

Plasmids can be sequenced and assembled from bacterial isolates or from complex microbial communities (metagenomically assembled plasmids). Since they originate from diverse sources and hosts, databases vary significantly in the traits they capture. Consequently, databases composed primarily of plasmids from bacterial isolates, often of clinical origin, are likely to capture a plasmid population shaped by the selective pressures on hosts, including antibiotic treatment. In contrast, databases built from metagenomes, often originating from environmental samples, reflect the plasmidomes of complex, native microbial communities. We hypothesize that plasmids assembled from isolates, therefore likely enriched with clinical specimens, and those assembled from metagenomes, thus representing a broad diversity of environmental species, are distinct. It is fundamental to understand plasmid ecology across different environments, but there is a significant knowledge gap: recent advances in metagenomics have allowed for the assembly of plasmids directly from environmental samples, yet systematic comparisons between isolate-derived and metagenome-assembled plasmids remain scarce. This gap is important because, most likely, clinical settings select for antibiotic resistance, whereas environmental communities experience diverse and less human-driven selective forces. Understanding how these different selective pressures shape plasmid characteristics will help to assess the true reservoir of antimicrobial resistance in natural environments or predict the evolutionary trajectories of clinically relevant plasmids.

An important characteristic that directly influences the epidemic potential of plasmids is their ability to transfer between bacterial cells. Conjugative plasmids (pCONJ) can transfer themselves by replication between bacterial cells, often between different strains or species. Therefore, several works have emphasized their pivotal role in bacterial evolution by acquiring clinically significant traits [9,10,11,12,13]. pCONJ are self-transmissible because they encode a complete gene set necessary for conjugation, namely a relaxase (MOB), an endonuclease, that cuts one of the DNA strands at the *nic* site (a nick site) of the respective origin of transfer—*oriT*, and the complete machinery for mating pair formation (MPF), which connects donor and recipient cells while serving as a channel for the plasmid transfer [14,15]. Mobilizable plasmids (pMOB) lack part of or all MPF genes, so, for transfer, they must hijack these genes’ products from other co-resident conjugative plasmids. Plasmids lacking relaxases but containing *oriT* are also mobilizable and belong to the pMOB group [3]. Finally, non-transmissible plasmids (pNT) lack an identifiable *oriT* locus and the genes mentioned above, suggesting they cannot transfer horizontally. However, these plasmids may yet harbor additional unidentified *oriT* [3,16]. Previous studies estimated that about one-quarter of the plasmids are pCONJ: 23% in a database comprising 11,386 plasmids from *E. coli* and *S. aureus*; and 25.2% in a database comprising 14,029 plasmids [10,17]. These studies were performed using plasmid databases likely of clinical interest, as they have been isolated from human pathogens or confer drug resistance. Therefore, databases generated from these data may not represent a random sample and may not be representative of the natural diversity of plasmids.

To further test whether plasmids assembled from clinical/isolates and those from metagenomes are subjected to different selective pressures, we conducted another comparative analysis of plasmids, this time involving only the plasmids found in the ESKAPEE group of pathogens (*Enterococcus faecium*, *S. aureus*, *Klebsiella pneumoniae*, *Acinetobacter baumannii*, *Pseudomonas aeruginosa*, *Enterobacter* spp., and *Escherichia coli*) [18]. These pathogens are responsible for the majority of nosocomial infections globally and have been designated as priority pathogens for which new antibiotics are urgently needed. Importantly, ESKAPEE organisms occupy a unique ecological position: they are found both as opportunistic pathogens in clinical settings and as environmental residents in soil, water, and wastewater systems [19,20]. This dual ecology makes them ideal sentinels for understanding how environmental reservoirs contribute to clinical antibacterial resistance. With the comparing of plasmids from ESKAPEE species across clinical/isolate and environmental/metagenome datasets, we can directly test whether the elevated resistance profiles observed in clinical isolates reflect intrinsic properties of these bacterial species or result from selective pressures in healthcare environments. If environmental populations of ESKAPEE pathogens harbor fewer resistance genes than their clinical counterparts, it suggests that healthcare-associated selection is the main cause for carrying ARGs, and that interventions targeting clinical antibiotic use may be particularly effective. Conversely, if environmental ESKAPEE plasmids also carry high resistance burdens, it would indicate a broader environmental reservoir requiring different mitigation strategies.

This study addresses a critical knowledge gap by uncovering how different plasmids differ in terms of resistance genes, virulence genes, and plasmid mobility types according to their ecology and the database from which they are collected. We conduct the first large-scale comparative analysis of plasmid populations across three major databases representing distinct ecological contexts and sampling methodologies. We used the RefSeq database [21] and two datasets from the IMG/PR database [22], isolate-derived plasmids (IMG/PR (I)) and metagenome-assembled plasmids (IMG/PR (M), hence systematically studying over 200,000 plasmid sequences. Therefore, we can distinguish genuine biological differences driven by divergent selective forces from potential technical artifacts inherent to different sequencing and assembly approaches. This is a unique study due to three main reasons: (i) we provide the most comprehensive assessment to date of how plasmid origin (clinical/isolate versus environmental/metagenome) influences fundamental plasmid characteristics, including size, mobility, and cargo genes; (ii) we compare isolate-derived plasmids from two independent databases (RefSeq and IMG/PR (I)), hence validating findings and control for database-specific biases; (iii) we extend our analysis to plasmids within ESKAPEE pathogens across different ecological contexts, therefore revealing how the same bacterial species harbor strikingly different plasmid populations depending on their environment, a finding with direct implications for understanding the clinical-environmental transmission of antimicrobial resistance.

## 2. Results

This study aims to elucidate the differences between plasmids originating from different datasets populations from isolate and metagenomic sources in terms of their mobility and the presence of ARGs and VGs.

We analyzed three distinct datasets: plasmids from RefSeq and from isolates from IMG/PR, likely enriched in clinical isolates (henceforth referred to as clinical/isolates); and plasmids from IMG/PR metagenomes, enriched in environmental plasmids (henceforth referred to as environmental/metagenomic). Hereafter, we refer to these datasets as RefSeq, IMG/PR (I), and IMG/PR (M).

### 2.1. Mobility and Size of Plasmids

**Figure 1:**
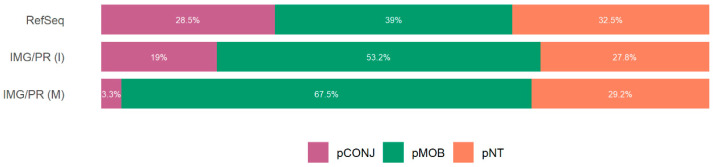
Proportion of plasmid mobility types within each dataset. The proportions of pCONJ, pMOB and pNT across the three datasets are statistically different (Chi-square test, χ^2^(4, n = 206,894) = 27,383, *p* < 2.2 × 10^−16^, with a medium effect size, Cramér’s V = 0.257, CI 95% = [0.254, 0.260]). This effect size implies that this difference in mobility type distribution is not only statistically significant but also biologically meaningful, not a statistical artifact of a large sample size. In RefSeq, pCONJ (adj. res. = 140.7) and pNT (adj. res. = 15.0) are observed more frequently than expected, whereas pMOB is underrepresented (adj. res. = −105.4). In IMG/PR (I), pCONJ also occurs more often than expected (adj. res. = 38.4), contrary to pMOB (adj. res. = −17.8) and pNT (adj. res. = −7.7). In IMG/PR (M), pMOB is overrepresented (adj. res. = 107.0), with pCONJ (adj. res. = −152.5) and pNT (adj. res. = −8.3) occurring less frequently than expected. pCONJ are represented in purple, pMOB in green, and pNT in orange.

Plasmids with all the necessary genes for transfer by conjugation (pCONJ) are more frequent among clinical/isolates (28.5% in RefSeq and 19% in IMG/PR (I)) than in plasmids of environmental/metagenome sources (only 3.3% in IMG/PR (M)) (Figure 1). In contrast, environmental plasmids are richer in pMOB (67.5%) than clinical/isolates plasmids (39.0% in RefSeq and 53.2% in IMG/PR (I)). Yet, there is almost no variation in the proportion of pNT plasmids: (32.5% in RefSeq, 27.8% in IMG/PR (I), and 29.2% in IMG/PR (M)) (Figure 1).

**Figure 2:**
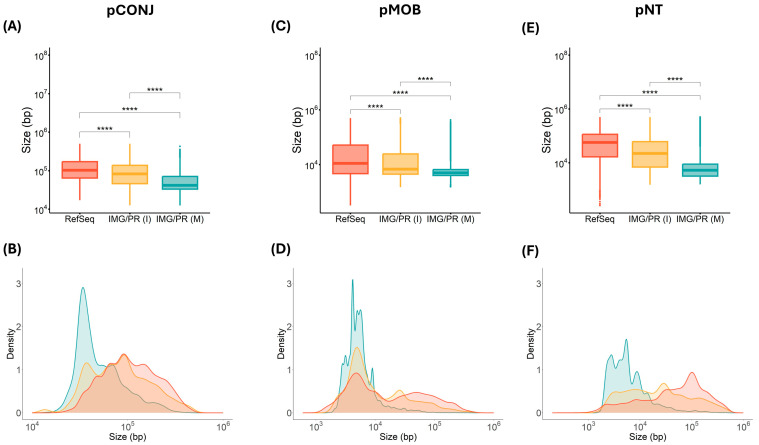
Plasmid size (bp) of the three plasmid mobility types across datasets. Orange indicates RefSeq samples, yellow indicates IMG/PR(I) samples, and blue indicates IMG/PR(M) samples. (**A**,**B**) pCONJ. Sizes of pCONJ across the three datasets differ (Kruskal–Wallis analyses: χ^2^(2, n = 24,052) = 3818, *p* < 2.2 × 10^−16^, η^2^ = 0.159, with a large effect size). A Dunn’s test revealed that pCONJ from Refseq are larger than pCONJ from IMG/PR (I) (Z = 20.3, *p*. adj < 2.2 × 10^−16^) and from IMG/PR (M) (Z = 61.5, *p*. adj < 2.2 × 10^−16^); and that pCONJ from IMG/PR (I) are larger than pCONJ IMG/PR (M) (Z = 34.3, *p*. adj < 2.2 × 10^−16^). (**C**,**D**) pMOB. Sizes of pMOB across the three datasets differ (Kruskal–Wallis analyses: χ^2^(2, n = 120,985) = 10,774, *p* < 2.2 × 10^−16^, η^2^ = 0.089, with a moderate effect size). A Dunn’s test revealed that pMOB from Refseq are larger than pMOB from IMG/PR (I) (Z = 16.9, *p*. adj < 2.2 × 10^−16^) and from IMG/PR (M) (Z = 94.3, *p*. adj < 2.2 × 10^−16^), and that pMOB from IMG/PR (I) are larger than that from IMG/PR (M) (Z = 57.8, *p*. adj < 2.2 × 10^−16^). (**E**,**F**) pNT. Sizes of pNT across the three datasets differ (Kruskal–Wallis analyses: χ^2^(2, n = 61,857) = 20,654, *p* < 2.2 × 10^−16^, η^2^ = 0.334, with a large effect size). A Dunn’s test revealed that pNT from Refseq are larger than pNT from IMG/PR (Z = 35.7, *p*. adj < 2.2 × 10^−16^) and from IMG/PR (M) (Z = 138.1, *p*. adj < 2.2 × 10^−16^), and that pNT from IMG/PR (I) are larger than pNT from IMG/PR (M) (Z = 68.9, *p*. adj < 2.2 × 10^−16^). (**B**,**D**,**F**) Density plots depict the size distribution for RefSeq, IMG/PR (I), and IMG/PR (M) datasets. The *x*-axis represents plasmid size (log scale) and the *y*-axis indicates relative density. Peaks highlight the most common size ranges within each dataset across the different plasmid mobility type. Figures showing densities normalize the area under the curve to one for each plasmid type. * *p* < 0.05, ** *p* < 0.01, *** *p* < 0.001, and **** *p* < 0.0001.

The median plasmid size for all mobility types is consistently higher across the two plasmid datasets representing the clinical/isolates than in the environmental/metagenomic plasmids (Figure 2).

The proportion of pCONJ in clinical/isolates (28.5% in RefSeq and 19% in IMG/PR(I)) is higher than in environmental/metagenome samples (3.3% in IMG/PR(M)). That is likely a consequence of the selection for horizontal transfer in healthcare settings (Figure 1). In contrast, smaller plasmids lacking part or all of the mating pair formation genes (pMOB) dominate in environmental communities (Figure 2).

### 2.2. Antimicrobial Resistance and Plasmid Mobility

**Figure 3:**
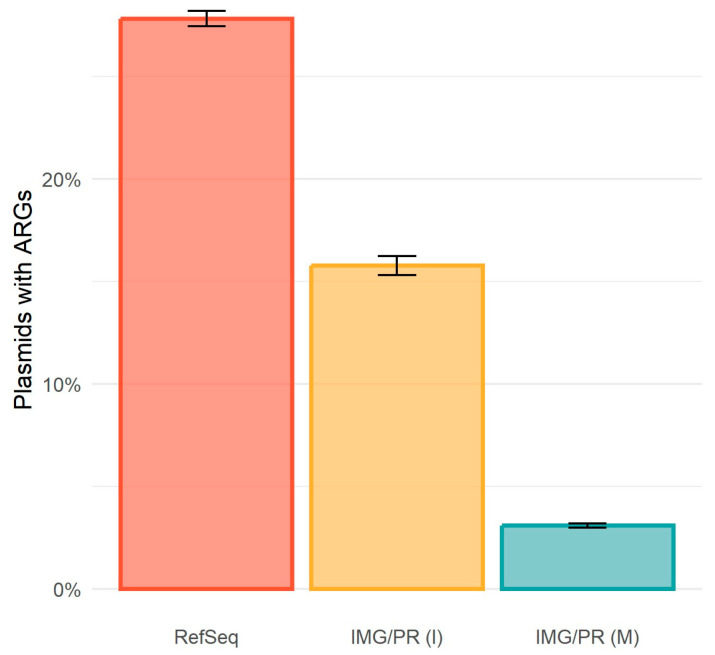
RefSeq contains 14,718 plasmids with ARGs (out of 52,909, hence 27.8% (CI 95% = [27.4%, 28.2%]) of the plasmids); IMG/PR (I) contains 3890 of 24,665 plasmids with ARGs (15.8%, CI 95% = [15.3%, 16.2%]), and IMG/PR (M) contains 4000 of 129,320 plasmids with ARGs (3.1%, CI 95% = [3.0%, 3.2%]). The proportion of plasmids with ARGs is different among the three datasets (Chi-square test: χ^2^(2) =22,608, *p* < 2.2 × 10^−16^, with a large effect size, Cramér’s V = 0.342, CI 95% = [0.338, 0.347]). This large effect size indicates the difference in ARG prevalence between clinical/isolate and environmental/metagenome plasmids (27.8% vs. 3.1%, a 9-fold difference) is not only statistically significant but also biologically meaningful. There are more plasmids with ARGs from RefSeq (adj. res. = 144.3) and IMG/PR (I) (adj. res. = 26.0) than expected, but fewer plasmids with ARGs from IMG/PR (M) than expected (adj. res. = −147.5).

To determine whether plasmids tend to carry more or fewer ARGs depending on their mobility type, and to assess whether the plasmid origin might influence this analysis, we performed a comprehensive screening of plasmids to detect ARGs using the AMRFinderPlus [23] database. Plasmids from clinical/isolates have a higher prevalence of ARGs (27.8% in RefSeq and 15.8% in IMG/PR (I)), than the environmental/metagenome ones (3.1%) (Figure 3).

**Figure 4:**
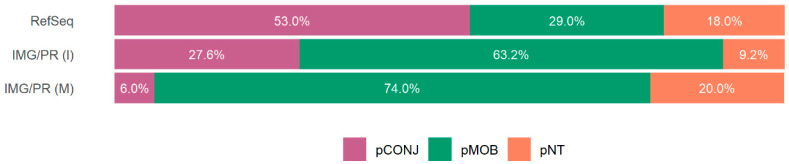
Distribution of plasmid mobility types among ARG-carrying plasmids in the three datasets. For each dataset, only plasmids carrying ARGs were considered. The figure shows the pCONJ, pMOB, and pNT percentages. In RefSeq plasmids, the association between plasmid mobility type and the presence of ARGs is statistically significant (Chi-square test: χ^2^(2) = 6131, *p* < 2.2 × 10^−16^, with a large effect size, Cramér’s V = 0.340, CI 95% = [0.332, 0.349]). There are more pCONJ with ARGs than expected (adj. res. = 77.5), but fewer pMOB with ARGs than expected (adj. res. = −29.3), and fewer pNT with ARGs than expected (adj. res. = −44.2). Among IMG/PR (I) plasmids carrying ARGs, the association between plasmid mobility type and the presence of ARGs is also statistically significant (Chi-square test: χ^2^(2) = 842, *p* < 2.2 × 10^−16^, although with a small effect size, Cramér’s V = 0.185, CI 95% = [0.176, 0.195]). There are more pCONJ with ARGs than expected (adj. res. = 15.0), and more pMOB with ARGs than expected (adj. res. = 13.6), but fewer pNT with ARGs than expected (adj. res. = −28.2). Although statistically significant, the small effect size suggests that the association between mobility type and ARG presence, while real, is modest in biological magnitude compared to RefSeq. Among IMG/PR (M) plasmids carrying ARGs, the association between plasmid mobility type and the presence of ARGs is also statistically significant (Chi-square test: χ^2^(2) = 234, *p* < 2.2 × 10^−16^, but with a very small effect size, Cramér’s V = 0.043, CI 95% = [0.037, 0.049]). There are more pCONJ with ARGs than expected (adj. res. = 9.6), and more pMOB with ARGs than expected (adj. res. = 8.9), but fewer pNT with ARGs than expected (adj. res. = −13.0). pCONJ are represented in purple, pMOB in green, and pNT in orange. Despite statistical significance due to the very large sample size (n = 129,320), the very small effect size indicates that this association has minimal biological importance in environmental plasmids.

The distribution of plasmid mobility types among ARG-carrying plasmids in the three datasets is represented in Figure 4. There are more pCONJ in clinical/isolates (53.0% in RefSeq and 27.6% in IMG/PR (I)) than in the environmental/metagenome’s plasmids (6.0%). However, the environmental/metagenome’s plasmids have more pMOB (74.0%) and pNT (20.1%) than the clinical/isolates (29.0% in RefSeq and 63.2% in IMG/PR (I) of pMOBs, and 18.0% in RefSeq and 9.2% in IMG/PR (I) of pNTs) (Figure 4).

The prevalence of ARGs is about 9-fold higher in clinical/isolates plasmids (27.8%) than in environmental/metagenome samples (3.1%). This difference probably results from antibiotic use in healthcare settings. The association between pCONJ and ARG carriage in the three datasets suggests that conjugative plasmids promote the transfer of resistance genes. In contrast, environmental microbiomes experience lower antibiotic exposure, where the fitness cost of maintaining resistance may outweigh survival benefits, leading to lower ARG prevalence.

As metagenomic samples exhibit greater genetic diversity, and the presence of distant orthologs necessitates the use of more permissive thresholds to detect resistance genes, different identity thresholds were used to detect resistance genes in samples from isolates and metagenomes. To account for this, we varied the identity thresholds in our analyses regarding plasmids assembled from metagenomes. Our findings indicate that these variations had minimal impact on ARG proportions. We identified ARGs in 3.52% of plasmids with 70% identity and 3.09% with 90% identity. Additionally, the percentage of resistance genes detected remains relatively consistent across different plasmid mobility types (Table 1).

### 2.3. Antibiotic Resistance in ESKAPEE Pathogens

**Figure 5:**
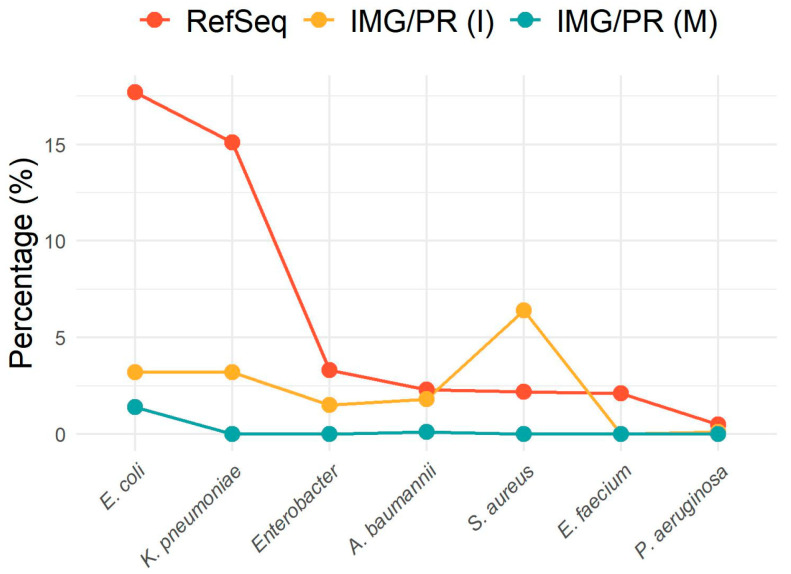
Percentage of plasmids from ESKAPEE pathogens across datasets. Relative abundance (%) of plasmids from the species belonging to the ESKAPEE group among plasmid hosts in each dataset. The *x*-axis displays species; the *y*-axis indicates the percentage of plasmids associated with each species. Red, yellow, and blue lines represent RefSeq, IMG/PR (I), and IMG/PR (M), respectively.

Antibiotic resistance levels are of particular importance when analyzing clinically important pathogens. We studied plasmids from pathogens belonging to the group of bacterial species known as ESKAPEE, comprising *A. baumannii*, *Enterobacter* sp., *E. faecium*, *Escherichia coli*, *K. pneumoniae*, *P. aeruginosa*, and *S. aureus* species [24]. There are more plasmids from species belonging to the ESKAPEE group in the clinical/isolate plasmids than in the environmental/metagenome plasmids (Figure 5).

The percentage of plasmids carrying ARGs among ESKAPEE pathogens is consistently higher in the clinical/isolates plasmids than in the environmental/metagenome one (Table 2).

The near-absence of ARGs in environmental/metagenome ESKAPEE plasmids (0 to 10.7% across species) compared to clinical counterparts (30.9 to 71.2%) indicates that high resistance in these priority pathogens is predominantly acquired through healthcare-associated selection rather than representing intrinsic species properties. This suggests environmental ESKAPEE populations are not direct sources of fully multidrug-resistant pathogens, but rather represent susceptible hosts that acquire resistance after clinical exposure.

### 2.4. Virulence and Plasmid Mobility

**Figure 6:**
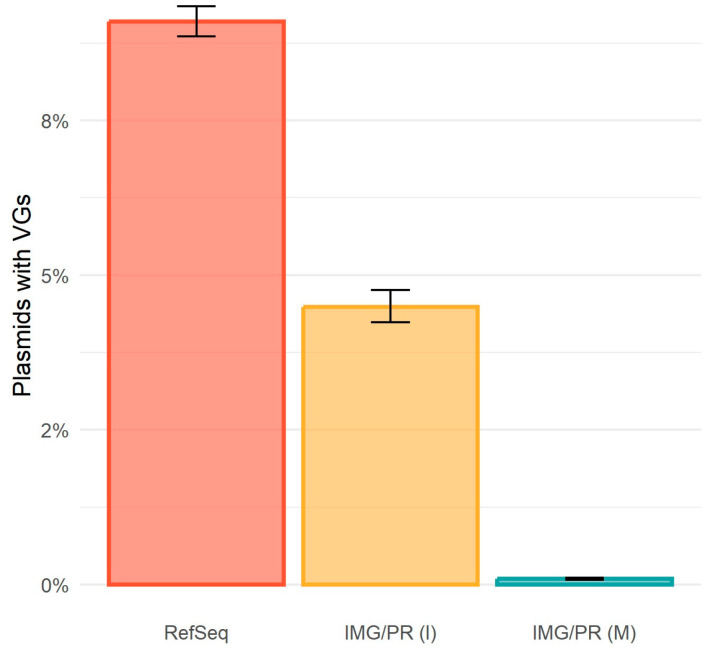
Proportion of plasmids with VGs in the three datasets. RefSeq contains 4814 plasmids with VGs (out of 52,909, hence 9.1% of the plasmids, CI 95% = [8.9%, 9.3%]), IMG/PR (I) contains 1107 plasmids with VGs (out of 24,665, hence 4.5% of the plasmids, CI 95% = [4.2%, 4.8%]), and IMG/PR (M) contains 116 plasmids with VGs (out of 129,320, hence 0.09% of the plasmids, CI 95% = [0.07%, 0.11%]). The proportion of plasmids with VGs differs across the three datasets (χ^2^(2) =11,001, *p* < 2.2 × 10^−16^, with a medium effect size, Cramér’s V = 0.231, CI 95% = [0.227, 0.234]). The medium effect size combined with the 100-fold difference in VG prevalence (9.1% vs. 0.09%) supports that this is a biologically meaningful distinction between clinical and environmental plasmid populations. There are more plasmids from RefSeq with VGs (adj. res. = 97.9) and from IMG/PR (I) (adj. res. = 15.6) than expected, but fewer plasmids with VGs from IMG/PR (M) than expected (adj. res. = −98.7).

We would also expect plasmids of clinical origin to carry more virulence genes, contributing to the pathogenicity of their hosts and supporting their coexistence with other bacterial pathogens in clinical environments. Our results corroborate this expectation: across all three datasets, the percentage of plasmids with VGs was low yet much lower in the environmental/metagenome plasmids (0.09% in the IMG/PR (M)) than in the clinical/isolates (9.1% in RefSeq and 4.5% in IMG/PR (I)) (Figure 6).

**Figure 7:**
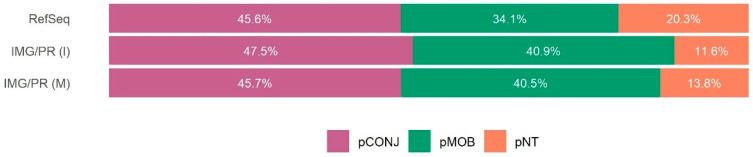
Distribution of plasmid mobility types among VG-carrying plasmids in the three datasets. For each dataset, only plasmids carrying VGs were considered. The figure shows the pCONJ, pMOB, and pNT percentages among these VG-carrying plasmids. The pCONJ plasmids are represented in purple, pMOB in green, and pNT in orange. Among RefSeq plasmids carrying VGs, the association between plasmid mobility type and the presence of VGs is statistically significant (χ^2^(2) = 814.6, *p* < 2.2 × 10^−16^, although with a small effect size, Cramér’s V = 0.124, CI 95% = [0.116, 0.135]). There are more pCONJ (adj. res. = 27.5) with VGs than expected, but fewer pMOB (adj. res. = −7.34) and pNT (adj. res. = −18.9) with VGs than expected. Yet, although statistically significant, the small effect size suggests that the preferential association of VGs with pCONJ is modest. Among IMG/PR (I) plasmids carrying VGs, 47.5% are pCONJ, 40.9% are pMOB, and 11.6% are pNT. In this dataset, the association between plasmid mobility type and the presence of VGs is statistically significant (χ^2^(2) = 640, *p* < 2.2 × 10^−16^, and with a small effect size, Cramér’s V = 0.161, CI 95% = [0.146, 0.177]). There are more pCONJ with VGs than expected (adj. res. = 24.8), but fewer pMOB (adj. res. = −8.4) and pNT (adj. res. = −12.3) with VGs than expected. Although the association is significant, the small effect size indicates that this association is biologically modest. Among IMG/PR (M) carrying VGs, 45.7% are pCONJ, 40.5% are pMOB, and 13.8% are pNT. In this dataset, the association between plasmid mobility type and the presence of VGs is statistically significant (χ^2^(2) = 654, *p* < 2.2 × 10^−16^, with a very small effect size, Cramér’s V = 0.071, CI 95% = [0.054, 0.089]). There are more pCONJ with VGs than expected (adj. res. = 25.6), but fewer pMOB (adj. res. = −6.2) and pNT (adj. res. = −3.7) with VGs than expected. However, here, the very small effect size indicates that this association has minimal biological importance, with statistical significance likely driven by the large sample size.

The distribution of plasmid mobility types among VG-carrying plasmids in the three datasets is represented in Figure 7. In general, all datasets show similar percentages of plasmids across mobility types. There are more pCONJ, followed by pMOB, and the least observed are pNT (Figure 7).

The prevalence of VGs is about 100-fold higher in clinical/isolate plasmids (9.1%) than in environmental/metagenome plasmids (0.09%), suggesting the existence of selective pressures favoring for the presence of virulence factors. The association between pCONJ and VGs indicates virulence that, like resistance, is mobile and can spread horizontally, a critical concern for the emergence of hypervirulent multidrug-resistant strains. The absence of VGs in environmental plasmids suggests these host-specialized traits provide no fitness advantage in non-clinical microbial communities.

### 2.5. Putative Bias in Metagenomic Data

One limitation of studying plasmids assembled from metagenomes is that it favors small plasmids. Because of their high copy number and simpler structure, these plasmids are often more easily recovered in metagenomic assemblies [25,26]. In contrast, large plasmids, which often contain repetitive and complex regions, are more prone to mis-assembly, frequently breaking into multiple fragments that may be mistakenly identified as several smaller, false plasmids. Therefore, we analyzed the percentage of plasmids carrying resistance genes by incrementally removing 10 to 70% of the smallest plasmids in the IMG/PR (M) dataset. Naturally, this changes the proportions of each plasmid mobility type in the sample (Table 3). Considering only the 30% largest plasmids, the percentage of pCONJ increases from 3.30% to 11.01%, remaining substantially below clinical/isolates (19 and 28.5%). This confirms assembly bias is not the primary driver of observed differences, validating the biological validity of clinical/isolate-environmental/metagenome comparisons. The slight increase (from 3.30% to 11.01%) was expected given that pCONJ must encode all the necessary enzymes for conjugation. Unexpectedly, however, the percentage of pNT also increases. Meanwhile, the percentage of pMOB decreases (Table 3).

However, this analysis resulted in almost no change in the percentage of plasmids with ARGs, with 3.09% when the entire sample was considered and 5.53% after removing 70% of the smallest plasmids (Table 4). That implies that ARGs are mostly present in bigger plasmids, in line with the previous results [2].

Even after removing the 70% smallest plasmids, the prevalence of ARGs remained low in plasmids from metagenomes. This corroborates the hypothesis that the low frequency of plasmids containing ARGs is not an artifact of plasmid assembly. However, the observation that ARGs are mostly present in larger plasmids suggests that technical limitations of plasmid assembly from metagenomes may underestimate the prevalence of ARGs in larger plasmids.

## 3. Discussion

This work aimed to uncover how plasmids vary in their content of resistance genes, virulence genes, and plasmid mobility types, including assessing the potential risk of epidemic dissemination of antibiotic resistance genes and virulence factors. For this purpose, we considered plasmids originating from bacterial isolates in the RefSeq and IMG/PR (I) datasets to overrepresent plasmids from clinical isolates; therefore, we refer to them as clinical/isolates for simplicity. In contrast, plasmids from the IMG/PR (M) dataset derive from metagenomic samples of diverse origins and, in this work, are designated as environmental/metagenome.

The comparative analysis performed here reveals that the plasmid populations inhabiting clinical/isolate bacteria are distinct from those circulating in environmental/metagenomes. These differences in key plasmid features, including size, antimicrobial resistance, virulence, and mobility, point to divergent evolutionary paths and ecological functions.

Plasmids from RefSeq are enriched in pCONJ. On the other hand, the environmental/metagenome plasmids exhibit a strong predominance of pMOB and an underrepresentation of pCONJ. Could the lower number of conjugative plasmids be due to the fragmentation of large plasmids into smaller pieces during sequencing and assembly, leading to an overrepresentation of false small plasmids? Our results suggest that this is not the case. After eliminating a large proportion of the smaller plasmids from the IMG/PR (M) dataset, keeping only the largest 30% of plasmids, the percentage of pCONJ is 11.01% (Table 3). This value remains lower than the percentage of pCONJ observed in clinical/isolates (19.0% in IMG/PR (I) and 28.6% in RefSeq) and in previous estimates reported in other works (approximately 25%) [3,10,17,27].

Regarding antimicrobial resistance, the proportion of ARGs in the clinical/isolate plasmids (27.8% in RefSeq and 15.8% in IMG/PR (I)) is higher than in the environmental/metagenome’s plasmids (only 3.1% in the IMG/PR (M)). The elevated ARG prevalence in RefSeq and IMG/PR (I) may indicate a sampling bias toward bacterial genomes from clinically relevant strains subjected to higher selective pressures to maintain antimicrobial resistance. This may also be a consequence of a focus on pathogens. In contrast, plasmids in the IMG/PR (M) dataset originate from more unbiased sampling efforts. Environmental and commensal bacteria may experience less intense selective pressures from antibiotics, leading to a lower prevalence of clinically relevant resistance genes. This, combined with technical limitations in metagenomic assembly and annotation, results in a plasmid population with a markedly different resistance profile.

Both databases used in this study, RefSeq and IMG/PR, include genomes generated using a variety of sequencing technologies and platforms, ranging from short-read, high-accuracy approaches (e.g., Illumina and Ion Torrent) to long-read technologies with lower per-base accuracy (e.g., Oxford Nanopore and PacBio). Genome assemblies derived from these different methodologies can vary substantially in the size of the reads, which in turn affects the ability to assemble and circularize DNA molecules such as plasmids. This technological heterogeneity may therefore influence the population structures inferred from the data. However, in this study, plasmids from each database were not stratified by sequencing methodology, and therefore, we cannot assess whether this influenced the comparisons between plasmids from isolates and metagenomes.

Interestingly, among plasmids encoding antibiotic resistance, pCONJ are always statistically significant above expected in all datasets, suggesting that antimicrobial resistance is linked to conjugation (Figure 4). This can be explained by their ability to move between cells, as conjugative plasmids act as vehicles for transferring antibiotic resistance genes. Over time, under selective pressures, such as antibiotic exposure, these plasmids are favored, allowing them and their bacterial hosts to thrive and spread within microbial communities. Therefore, this result aligns with the nature of conjugative plasmids, once their intrinsic capacity of mobility and the genetic elements they usually carry (such as insertion sequences) facilitate the acquisition and dissemination of resistance genes.

Due to the nature of the samples, the clinical/isolate plasmids contain a higher proportion of plasmids belonging to the ESKAPEE group of pathogenic bacteria. Interestingly, the environmental/metagenome plasmids of ESKAPEE pathogens harbor a low percentage of ARGs (Figure 5). This result may indicate that the high levels of resistance detected in these organisms in clinical isolates, as well as the associated risk, may be due to the selective pressure they are exposed to in medical contexts.

Regarding virulence, the pattern is similar to the one observed with ARGs. We detected a higher percentage of VGs in clinical/isolates (9.1% in RefSeq and 4.5% in IMG/PR (I)) than in the environmental/metagenome plasmids (0.09% in IMG/PR (M)). The number of plasmids with identified VGs was very low across all datasets (Figure 6), particularly in IMG/PR (M) (n = 116, 0.09%). The assumptions of the chi-square test were verified, namely all expected frequencies above five. However, the low absolute counts require cautious interpretation. The scarcity may reflect genuine biological patterns (virulence factors providing limited advantage outside host contexts), but could also stem from the following: (i) annotation limitations, as VGs may be misclassified or share homology with other systems (e.g., conjugation machinery); (ii) metagenomic assembly challenges for VG-containing regions; (iii) database bias toward well-characterized clinical virulence factors, leading to under detection of divergent environmental homologs. Therefore, conclusions regarding VGs should be considered preliminary pending improved annotation tools and experimental validation.

## 4. Materials and Methods

Plasmid sequences from IMG/PR database were obtained on 19 December 2023 (https://genome.jgi.doe.gov/portal/IMG_PR/IMG_PR.home.html) [22]. We selected plasmids classified as putatively complete and removed all plasmids larger than 500 kb that could represent potential secondary chromosomes, which are typically comparable in size to the main chromosome, carry essential housekeeping genes, and are indispensable for cell viability. Unlike plasmids, which are generally dispensable and encode accessory functions, secondary chromosomes have evolved to fulfill core cellular roles [28]. We retained plasmids identified across isolate genomes (24,665 sequences) and metagenomes (129,320 sequences), resulting in 153,985 plasmids. We downloaded 54,264 complete plasmids from the NCBI RefSeq database on February 2025 [21]. We also removed plasmids larger than 500 kb for this dataset, retaining 52,909 plasmids.

ARGs were detected using AMRFinderPlus version 4.0.19, database version 2025-03-25.1, with default parameters [23]. VGs were detected using ABRicate v1.0.1 against the Virulence Factors Database (VFDB) (parameters: ‘--db vfdb’), which includes 4360 sequences [29]. VFDB was last updated on 1 February 2024. AMRFinderPlus identifies resistance genes with a default minimum amino-acid sequence identity of 90%. To analyze the robustness of the results in the metagenomic data (IMG/PR (M) dataset), we varied the identity values to 70% and 80%. Metagenomic datasets are typically enriched in small plasmids. To evaluate the impact of these plasmids on the distribution of various mobility types and the proportion of plasmids carrying ARGs, we gradually eliminated a certain percentage of the smallest plasmids. First, we excluded the smallest 10% plasmids (number of base pairs), then 20%, 30% and so on up to 70%, thereby retaining only the largest 30%.

To identify conjugative systems in RefSeq plasmids, we applied MacSyFinder v2.1.3 [27] with the parameters --models CONJScan/Plasmids all --db-type ordered_replicon, following the identification method used in the IMG/PR database. Plasmids encoding a conjugative element (T4SS) were classified as pCONJ. If no T4SS was detected but a mobilization system (MOB) was present, plasmids were classified as pMOB. Plasmids lacking both T4SS and MOB genes were subsequently screened for the presence of an origin of transfer (*oriT*). Those carrying an *oriT* were also assigned to the pMOB group, whereas plasmids without any of these features were considered pNT. To detect *oriTs*, we used a curated database of 91 experimentally validated sequences [29] and performed a blastn v2.14.0+ search with the parameters -task blastn-short -evalue 0.01 [30]. Hits were retained only when both identity and coverage exceeded 80%.

The IMG/PR database [22] contains metadata regarding the presence of mobility genes, type IV coupling protein (T4CP), type IV secretion systems (T4SS), mating pair formation (MPF), obtained using CONJscan HMM models [31], and origin of transfer (*oriT*) obtained against a database of *oriT* sequences [3]. We classified plasmids as pCONJ, pMOB, or pNT based on the machinery required for DNA transfer. Plasmids encoding mobility genes, T4CP, T4SS, and MPF system were classified as pCONJ. If plasmids were not included in the pCONJ category, they were classified as pMOB if they contained mobility genes or an *oriT*. The remaining plasmids were pNT. Nevertheless, these non-transmissible plasmids may have an unidentified *oriT*, owing to the limited understanding of these regions [3].

The species where the plasmids were identified were already mentioned and validated in the two databases used in this study [32].

### Statistics

We performed the statistical analyses in R, version 4.4.2 [33]. We used Pearson’s chi-squared test to analyze the dependence between two categories and the respective residuals, measuring effect size using Cramér’s V from rcompanion package, version 2.4.36. Effect sizes were interpreted using Cohen’s conventions: Cramér’s V < 0.1 (very small), 0.1–0.3 (small to medium), 0.3–0.5 (medium to large), and >0.5 (large). For 2 × 3 contingency tables (mobility types across datasets), the following adjusted thresholds were applied: V < 0.07 (negligible), 0.07–0.21 (small), 0.21–0.35 (medium), >0.35 (large).

We used Dunn’s post hoc test from FSA package version 0.9.5 with Bonferroni correction for multiple comparisons to identify which groups differ. Visualizations were generated with the R package ggplot2, version 3.5.1.5.

## 5. Conclusions

Our results revealed significant differences plasmids populations. Plasmids from clinical/isolates sources are larger, more often conjugative, and carry a higher number of antimicrobial resistance (ARG) and virulence (VG) genes than environmental/metagenomes ones. This pattern holds even for ESKAPEE pathogens, where environmental/metagenome plasmids carry far fewer ARGs than the clinical/isolate ones. These contrasts likely reflect biological differences, namely different selective conditions.

Although assembling plasmids from metagenomes avoids the selective pressures associated with strain isolation and provides a more representative distribution, it may also increase the risk of false negatives by assembling truncated molecules [34] and still fail to capture the full diversity of plasmids. This highlights the need for caution when drawing conclusions based on specific datasets, as sample type and dataset content can strongly influence outcomes.

## Figures and Tables

**Table 1:** Percentage of each plasmid mobility type carrying ARGs considering different identity thresholds in the IMG/PR (M) dataset.

Identity (%)	Total (%)	pCONJ (%)	pMOB (%)	pNT (%)
90	3.09	5.60	3.39	2.12
80	3.45	6.00	3.89	2.16
70	3.52	6.14	3.98	2.20

**Table 2:** Proportion of plasmids carrying ARGs in each dataset, by ESKAPEE pathogens. Adjusted residuals were calculated using the Chi-squared test. NA denotes values that were not assigned.

Dataset	ESKAPEE Pathogens	Number of Plasmids	Number of Plasmids with ARGs	Percentage of Plasmids with ARGs	Adjusted Residuals
RefSeq	*A. baumannii*	1204	372	30.9	1.4
IMG/PR (I)		456	161	35.3	3.0
IMG/PR (M)		127	0	0	−7.6
RefSeq	*Enterobacter* sp.	1744	764	43.8	5.0
IMG/PR (I)		358	106	29.6	−5.0
IMG/PR (M)		0	0	0	NA
RefSeq	*E. faecium*	1116	429	38.4	NA
IMG/PR (I)		0	0	0	NA
IMG/PR (M)		0	0	0	NA
RefSeq	*E. coli*	9343	3746	40.1	33.7
IMG/PR (I)		777	117	15.1	−10.7
IMG/PR (M)		1773	5	0.3	−31.4
RefSeq	*K. pneumoniae*	7977	4223	52.9	0.2
IMG/PR (I)		790	427	54.1	0.7
IMG/PR (M)		28	3	10.7	−4.5
RefSeq	*P. aeruginosa*	285	128	44.9	2.4
IMG/PR (I)		36	17	47.2	0.7
IMG/PR (M)		25	0	0	−4.4
RefSeq	*S. aureus*	1157	719	62.1	−5.0
IMG/PR (I)		1590	1132	71.2	5.0
IMG/PR (M)		1	1	100	0.7

**Table 3:** Percentage of each plasmid mobility type, removing different slices of the smaller plasmids in the IMG/PR (M) dataset.

Plasmids Remaining (%)	Number of Plasmids	Mean Length	Minimum Length	pCONJ (%)	pMOB (%)	pNT (%)
100	129,320	9996.8	1546	3.30	67.45	29.25
90	116,388	10,831.1	2834	3.67	69.63	26.70
80	103,456	11,795.0	3463	4.13	69.67	26.20
70	90,524	12,933.2	4138	4.72	69.11	26.17
60	77,592	14,375.3	4551	5.50	66.59	27.91
50	64,660	16,275.3	5165	6.60	63.63	29.77
40	51,728	18,979.9	5697	8.25	62.67	29.07
30	38,796	23,270.7	6693	11.01	55.21	33.78

**Table 4:** Percentage of each plasmid mobility type carrying ARGs, removing different slices of the smaller plasmids in the IMG/PR (M) dataset.

Plasmids Remaining (%)	Number of Plasmids	Mean Length	Minimum Length	Plasmids with ARGs (%)	pCONJ (%)	pMOB (%)	pNT (%)
100	129,320	9996.8	1546	3.09	5.60	3.39	2.12
90	116,388	10,831.1	2834	3.27	5.60	3.56	2.22
80	103,456	11,795.0	3463	3.61	5.60	3.98	2.30
70	90,524	12,933.2	4138	4.04	5.60	4.49	2.56
60	77,592	14,375.3	4551	4.48	5.60	5.11	2.77
50	64,660	16,275.3	5165	5.09	5.60	6.00	3.02
40	51,728	18,979.9	5697	4.62	5.60	4.86	3.85
30	38,796	23,270.7	6693	5.53	5.60	6.21	4.38

## Data Availability

All data from this study are presented in the paper.
